# Asymmetric Cell Division and Tumor Heterogeneity

**DOI:** 10.3389/fcell.2022.938685

**Published:** 2022-07-04

**Authors:** Zizhu Li, Ying Yi Zhang, Haomiao Zhang, Jiaxuan Yang, Yongze Chen, Hezhe Lu

**Affiliations:** ^1^ State Key Laboratory of Membrane Biology, Institute of Zoology, Chinese Academy of Sciences, Beijing, China; ^2^ Institute for Stem Cell and Regeneration, Chinese Academy of Sciences, Beijing, China; ^3^ University of Chinese Academy of Sciences, Beijing, China; ^4^ Centre for Systems Biology, Lunenfeld-Tanenbaum Research Institute, Mount Sinai Hospital, Toronto, ON, Canada; ^5^ School of Stomatology, Dalian Medical University, Dalian, China; ^6^ College of Biological Sciences, China Agricultural University, Beijing, China

**Keywords:** asymmetric cell division, tumorgenesis, drug resisitance, symmetric cell division, tumor heterogeneity

## Abstract

Asymmetric cell division (ACD) gives rise to two daughter cells with different fates after mitosis and is a fundamental process for generating cell diversity and for the maintenance of the stem cell population. The cancer stem cell (CSC) theory suggests that CSCs with dysregulated self-renewal and asymmetric cell division serve as a source of intra-tumoral heterogeneity. This heterogeneity complicates the diagnosis and treatment of cancer patients, because CSCs can give rise to aggressive clones that are metastatic and insensitive to multiple drugs, or to dormant tumor cells that are difficult to detect. Here, we review the regulatory mechanisms and biological significance of asymmetric division in tumor cells, with a focus on ACD-induced tumor heterogeneity in early tumorigenesis and cancer progression. We will also discuss how dissecting the relationship between ACD and cancer may help us find new approaches for combatting this heterogeneity.

## Introduction

Stem cells are known for their ability to self-renew and to differentiate into different cell types (Morrison and Kimble, 2006). One strategy by which stem cells achieve these two goals is through a unique mode of division: stem cells can either replicate itself through symmetric cell division (SCD) or produce two daughter cells with different cell fates through asymmetric cell division (ACD) (Kahney et al., 2017). A balance between these two forms of division is essential for maintaining tissue homeostasis; failure to maintain homeostasis can lead to severe outcomes such as tumorigenesis (Neumüller and Knoblich, 2009).

*Cancer* stem cells (CSCs), a subpopulation of tumor cells that possess stem cell-like properties, have been identified in many tumor types. Accumulating evidence suggests that CSCs with dysregulated self-renewal and ACD give rise to tumor cells with a variety of properties and thus serve as a source of intra-tumoral heterogeneity (Lee et al., 2016). This heterogeneity complicates the diagnosis and treatment of cancer patients, because CSCs can generate tumor cell clones that are multi-drug-resistant, metastatic, or dormant, which makes them difficult to detect (Knoblich, 2010; Singh and Settleman, 2010; Viale et al., 2014). Here, we review the current understanding of ACD and discuss the relationship between ACD and tumor heterogeneity.

## Mechanisms of Asymmetric Cell Division

The basic mechanisms of ACD were initially explored in *Drosophila melanogaster*. The construction of the *Drosophila* nervous system is mediated by embryonic neuroblasts (NBs) through a series of ACD events: first of all, cellular components in NBs are distributed asymmetrically before mitosis, cell-fate-determining factors such as Numb、Brat (TRIM32 in vertebrates) and Prospero (PROX1 in vertebrates) are concentrated in the basal cell cortex, while the apical region expresses strong stemness signals, leading to unequal separation during cytokinesis (Bello et al., 2008; Bowman et al., 2008). As a result, ACD of NBs produces another NB and a more differentiated progenitor cell called ganglion mother cell (GMCs). Studies in *Drosophila* have also uncovered proteins involved in the establishment of cell polarity, including the polarity complex Par3/Par6/aPKC and the related protein WD40 protein lethal giant larvae (Lgl) (Lee et al., 2006a), as well as protein kinase Aurora-A (Wirtz-Peitz et al., 2008). Proteins necessary for polar coupling of mitotic spindle to the cell cortex have been identified, such as Pins (LGN in vertebrates), the Par3 binding protein Inscuteable, the heterotrimer G protein subunit G*α*i and the Dynein adapter Mud (NuMA in vertebrates) (Izumi et al., 2006; Santoro et al., 2016). Insights into the molecular mechanisms of ACD in the *Drosophila* model warrant further studies in vertebrates.

In vertebrates, molecular determinants of ACD are highly conserved; however, their modes of division may vary depending on the cell/tissue type. In recent years, advancements in research technologies, such as stem cell cultures, lineage tracing, cell imaging and molecular tracers, have largely facilitated the study of stem cell ACD in more complex mammalian systems, providing insights into the complexity of cellular and environmental asymmetry that play important roles in cell fate determination (Santoro et al., 2016). Studies so far suggest that ACD in mammalian stem cells is mediated by two different mechanisms: one is niche-dependent ACD, which is induced by external signals; the other is termed spontaneous ACD, which is determined by the differential distribution of proteins, RNA transcripts and macromolecules between two daughter cells.

**FIGURE 1:**
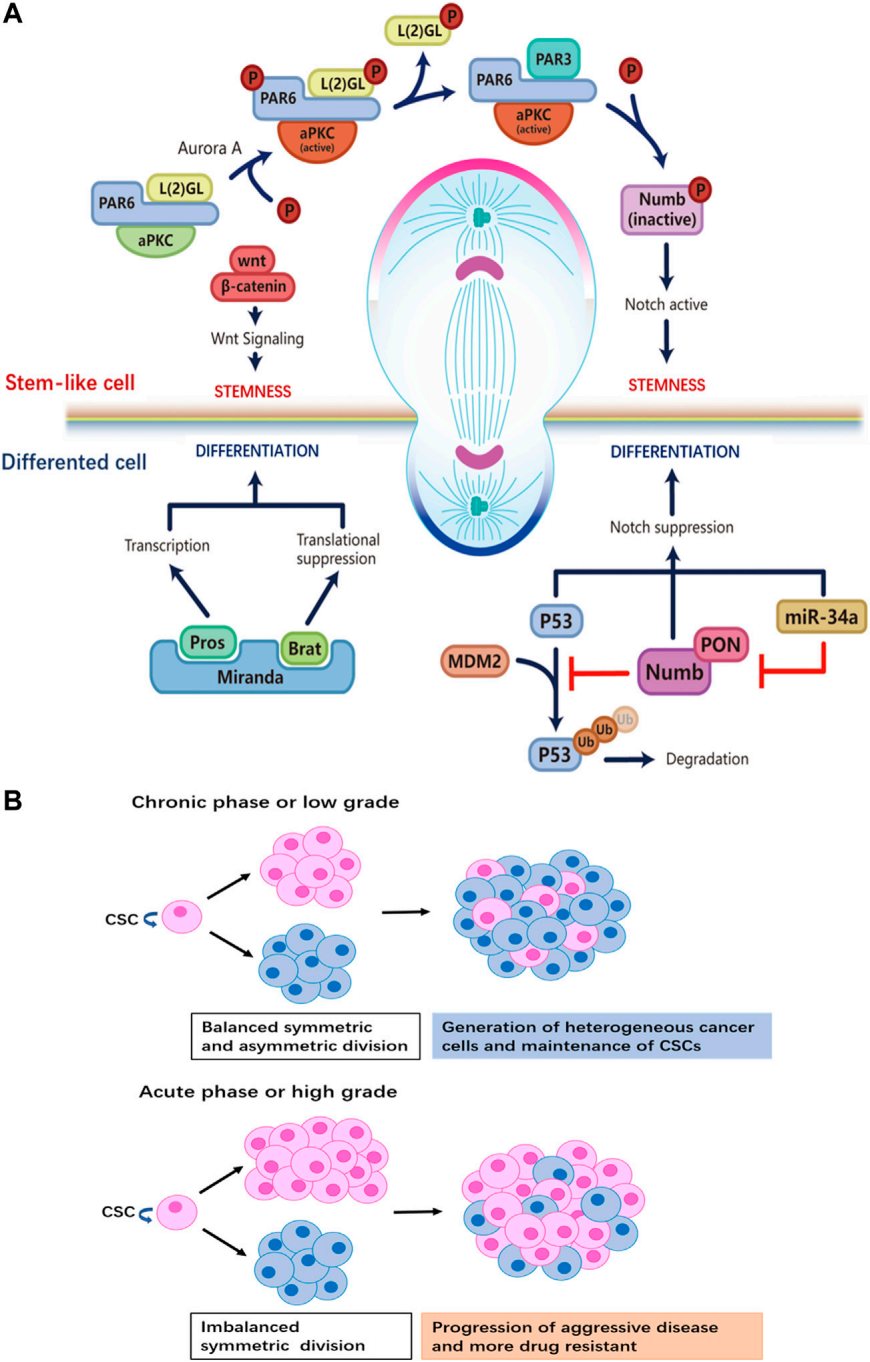
**(A)** Distinct regulatory machineries are involved in the two daughter cells during ACD of stem cells. Stem cell at the apical pole: formation of the aPKC/PAR6/PAR3 complex plays a crucial role for the establishment of the ‘self-renewal’ identity at the apical pole. Aurora-A protein kinase activates aPKC, which then phosphorylates L (2)GL; then L (2)GL is released from the complex and replaced by PAR3. The aPKC/PAR6/PAR3 complex phosphorylates Numb, releasing it from the apical membrane. PON brings Numb to the basal pole that will become the differentiated daughter cell. By removing Numb, Notch signaling is active to maintain the stemness of the daughter cell at the apical side. Wnt signaling also participates in promoting self-renewal in the daughter cell, though details of the mechanism are not known. Differentiated cell at the basal pole: the adapter protein Miranda recruits Prospero and Brat. As a translational repressor, Brat suppresses the synthesis of proteins necessary for proliferation. The transcription factor Prospero promotes the expression of genes that drive differentiation after Miranda is degraded. Ubiquitination-dependent degradation of P53 is inhibited by Numb, while the miR-34-Numb-Notch feedback loop suppresses Notch level and favors differentiation in the daughter cell. **(B)** The relationship between tumor progression and the ratio of CSCs that undergo ACD versus SCD. When SCD and ACD are balanced, tumor generates heterogeneity while maintaining the pool of CSCs. On the other hand, a switch from ACD to SCD in CSCs results in the expansion of the stem cell pool.

Multiple studies have revealed that the Notch signaling pathway plays a central role in instructing stem cell ACD (Srinivasan et al., 2016a; Rossi and Desplan, 2017). Numb negatively regulates Notch signaling, the asymmetric distribution of Numb is regulated by the Par3/Par6/aPKC complex; the assembly of the Par3/Par6/aPKC complex alters the substrate specificity of aPKC to phosphorylate Numb, resulting in its release from the apical cortex. Phosphorylated Numb is localized to the basolateral cell cortex with the help of an adapter protein, partner of Numb (PON), which is activated by the Polo kinase (Gómez-López et al., 2014). Notch signaling is restricted by the asymmetric distribution of Numb; Numb mediates ubiquitin-dependent degradation of the Notch receptor and blocks the nuclear translocation of Notch intracellular domain (NICD1) (Mcgill and Mcglade, 2003). In addition, studies have shown that the microRNA miR-34, suppresses Notch expression by directly binding to the 3′ untranslated region (UTR) of Notch mRNA; the asymmetric distribution of miR-34 results in distinct cell fates in the two daughter cells, acting as a bimodal switch between self-renewal and differentiation. Interestingly, miR-34 also inhibits Numb expression by binding to the 3′UTR of Numb mRNA. Thus, miR-34, Numb, and Notch form an incoherent feedforward loop (IFFL), which maintains the homeostasis of Notch level (Bu et al., 2016) (Figure 1A). This regulatory mechanism further fine-tunes Notch signaling and cell fate determination.

Wnt signaling is another key regulator of stem cell ACD (Varga and Greten, 2017). A Wnt ligand gradient in the stem cell niche has been shown to instruct the directed movement of centrosomes and mitotic spindles and mediate the differential distribution of downstream Wnt signaling molecules in daughter cells (Figure 1A). The daughter cell proximal to higher levels of Wnt expresses high levels of Wnt pathway genes including β-catenin and stemness-related genes, thereby maintaining the stemness of the daughter cell. On the contrary, the daughter cell distal to Wnt is destined to differentiate. ACD guided by Wnt in the stem cell niche ensures the orderly spatial distribution of stem cells and differentiated progenies.

Another molecular determinant of ACD is p53 (Santoro et al., 2016), a well-studied tumor-suppressor that induces cell-cycle arrest and apoptosis in cells with DNA mutations or damage. Importantly, p53 is involved in the maintenance of the stem cell pool by regulating the modality of cell division. In mammary gland epithelial cells, p53 expression induces a shift from exponential growth to linear growth, restricting the expansion of mammary epithelial cells by upregulating the ratio of stem cells that undergo ACD (Cicalese et al., 2009). Furthermore, Numb interacts with and stabilizes p53 by blocking the ubiquitination and degradation of p53 induced by the E3 ubiquitin ligase MDM2 (Tosoni et al., 2015; Kim and Ronai, 2018) (Figure 1A).

## Asymmetric Cell Division and Tumor Heterogeneity

### Asymmetric Cell Division and Cancer Stem Cell Heterogeneity in Tumorigenesis

In the early stages of tumorigenesis, normal cells acquire key driver mutations that confer a growth advantage, and undergo rapid clonal expansion (Gerdes et al., 2014). It has been shown that the aberrant expression of key regulators of ACD is an important contributor to early carcinogenesis. When NBs carrying loss-of-function mutations in key regulators of ACD were allografted into the abdomen of wild-type adult *Drosophila*, a rapid expansion and invasion of the mutant cell population into the host’s abdomen was observed; and the tumorigenicity of the mutant cells increased with subsequent passaging of the allografts, suggesting that uncontrolled proliferation due to dysregulation of ACD was one of the key factors in the tumorigenic transformation (Lee et al., 2006a; Betschinger et al., 2006; Lee et al., 2006b; Bowman et al., 2008). In mammalian models, a switch from ACD to SCD in stem cells triggers a severe disruption of tissue homeostasis and drives tumor formation (Klezovitch et al., 2004; Mccaffrey and Macara, 2009); regulatory factors of ACD are frequently found in lists of aberrantly expressed genes associated with cancer (Pece et al., 2004; Ito et al., 2010; Gómez-López et al., 2014). However, mutating this factor alone is not sufficient for tumor initiation in mammals (Iden et al., 2012; Mccaffrey et al., 2012).

It has been hypothesized that certain tumors originate from normal stem cells (Sell, 2007), but the mechanisms by which normal stem cells progressively undergo malignant transformation are not clear (Kasper, 2008; Sell, 2010). Recently, researchers have identified a link between the dysregulated division pattern of +4 stem cell (SC) in the gastric antrum and gastric carcinogenesis. +4 SCs, marked by expression of Cck2r [a G-protein coupled receptor (GPCR)] and Delta-like ligand 1 (DLL1), are Notch^low^/Numb^+^ cells that undergo ACD predominantly under normal conditions; and their proliferation is inhibited by signaling from gastrin-expressing endocrine cells. Studies in mouse models have shown that treatments with carcinogens lead to a down-regulation of gastrin secretion and as a result, + 4 SCs gradually up-regulate the proportion of cells that undergo symmetric division, leading to an expansion of the + 4 SC pool. The disruption in tissue homeostasis caused by the switch to symmetric division is thought to be closely related to gastric carcinogenesis (Chang et al., 2020). Another study has shown the involvement of ACD in early tumor formation in mutant K-Ras-induced spontaneous lung cancer model. The pre-cancerous adenoma cells initiated a positive CD44/Zeb1 feedback loop through nuclear polarization of key transcription factors during asymmetric division, generating an intermediate transitional population of Zeb1^hi^CD44^hi^ cells that are tumorigenic (Liu et al., 2018).

CSCs have been reported to control the ratio of cells that undergo ACD versus SCD during early tumorigenesis. In order to achieve rapid clonal expansion and establish survival advantage, CSCs enable self-renewal at a certain rate to preserve stemness, while generating differentiated cells to constitute a heterogeneous tumor. Table 1 summarizes recent reports on how ACD-related pathways/genes influence cancer progression (Zimdahl et al., 2014; Tosoni et al., 2015; Ali et al., 2016; Bai et al., 2016; Damodaran et al., 2017; Keysar et al., 2017; Mizukawa et al., 2017; Castro-Oropeza et al., 2018; Liu et al., 2018; Stypulkowski et al., 2018; Sugioka et al., 2018; Choi et al., 2020; Pajuelo-Lozano et al., 2020). Interestingly, the regulatory factors of ACD display heterogeneous expression: factors that direct the differentiation of daughter cells are often associated with loss-of-fuction mutations or downregulation; while factors that maintain the stemness are often overexpressed in tumors. These factors affect ACD and cell heterogeneity directly or indirectly through Notch or Wnt signaling pathway. For example, the palmitoacylase APT1 promotes the asymmetric localization of Numb and β-catenin on the plasma membrane by interacting with CDC42. APT1 contributes to the activation of Notch or Wnt signaling, while CDC42 restricts APT1 activity to only one of the two daughter cells, results in formation of heterogeneous cell population during ACD. APT1 and CDC42 cooperate to maintain a self-renewal stem cell pool; and loss of APT1 depletes a specific tumorigenic stem cell subpopulation (Stypulkowski et al., 2018). Another study has shown that in KRAS-mediated lung adenocarcinoma (LADC), the protein kinase Cι(PKCι) activates NOTCH3 expression by phosphorylating ELF3, which driving ELF3 recruitment to NOTCH3 promoter. The unequal distribution of PKCι results in a difference in NOTCH3 signaling levels between two daughter cells. This difference ultimately leads to ACD of tumor-initiating cells (TICs), preserving the TIC pool while generating heterogeneous populations (Ali et al., 2016). These discoveries suggest that ACD plays an important role in cell heterogeneity and cancer progression.

**TABLE 1:** ACD-related regulators and their role during cancer formation and progression.

Gene	ACD-related Pathway	Model	cancer Type	Dysregulation in Cancer	Influence on the division mode
NUMB-interacting protein (TBC1D15)	Numb-Notch1-Nanog、P53	mouse	Hepatocellular carcinoma (HCC)	overexpressed	promote symmetric renewal、promote stemness
MAD2	-	mouse	gastric carcinoma (GC)	overexpressed	promote stemness
Lnc34a	Notch、Wnt	mouse	colon cancer	overexpressed	promote symmetric renewal
CDC42	Wnt	mouse	triple receptor negative breast cancer (MDA-MB-231 cell line)、blood/acute myeloid leukemia	overexpressed	promote ACD
Aurora-A	Numb-Notch、p53	*Drosophila*	-	overexpressed	maintaince ACD、promote stemness
miR-34a	Numb-Notch	mouse	colon cancer	decreased expression	promote differentiation
CD44/Zeb1 loop	-	mouse	lung adenocarcinoma	express in cancer-generating cells	promote ACD
SOX2	-	mouse	Head and Neck Squamous *Cancer* Stem Cells	critical for propagation of CSCs	maintaince ACD
EGFL6	-	mouse	ovarian cancer	express in tumor vascular cells and in some cancer cells	induce ACD
PKCι	Notch	mouse	lung adenocarcinoma	overexpressed	drive ACD
APT1	Numb-Notch、wnt	mouse	MDA-MB-231 cell line	critical for propagation of CSCs	direct ACD
lis1	Numb-Notch	mouse	acute myelogenous leukemia (AML)	critical for propagation of CSCs	promote symmetric renewal
miR-200b-3p	Notch	mouse	pancreatic cancer	decreased expression	promote ACD
APC	Wnt/β-catenin	mouse	colon cancer	frequently mutated	direct ACD
numb	Numb-Notch	mouse	mammary carcinomas, lung cancer, chronic myeloid leukemia	decreased expression	promote differentiation
p53	Numb-Notch	mouse	Breast cancer	decreased expression	promote ACD

### Asymmetric Cell Division and Cancer Heterogeneity in Drug Resistance

CSCs are capable of responding rapidly and flexibly to environmental challenges, making CSCs a major source of drug-resistant tumor cells that give rise to disease recurrence after drug treatment (Cabrera et al., 2015; Batlle and Clevers, 2017). The transition of CSCs from asymmetric to symmetric renewal division increases the proportion of CSCs in a tumor, which is predictive of malignant progression of disease and increased difficulty in treatment (Lytle et al., 2018). Aside from being able to swiftly adapt and react to various external stresses, CSCs pose challenges to cancer therapy as they are a source of high intra-tumoral heterogeneity (Singh et al., 2015), which leads to different sensitivity to treatment in cancer patients (Donnenberg and Donnenberg, 2005) (Figure 1B).

It has been shown that tumor cells can asymmetrically divide to produce progenies that are slow-cycling and those that are fast-cycling. Fast-cycling cells rapidly expand to accelerate tumor progression, while slow-cycling cells have a relatively slower doubling rate and may survive chemotherapy targeting the rapidly proliferating population, therefore serving as an important reservoir of tumor-initiating cells post-treatment. Studies have demonstrated that after ACD, CSCs can produce dormant cells that retain labeled nucleotide or fluorescent lipid markers (Pece et al., 2010; Majumdar et al., 2020). In patients with breast cancer, ACD of fast-cycling cancer cells produced slow-cycling G0-like progenies that are AKT^low^Ros^low^Hes1^hi^, in a process that is dependent on the asymmetric inhibition of AKT/PKB kinase signals in the two daughter cells at the end of mitosis (Dey-Guha et al., 2011). In colorectal cancer (CRC), a subpopulation of tumor cells with stem cell properties, called colorectal cancer-initiating cells (CCIC), harbors high internal heterogeneity. There are two types of CCICs ——MYC-dependent, fast-cycling cells expressing LGR5, CD133, and CD44, and slow-cycling cells expressing BMI1, hTERT, and HOPX. It was found that the 2 cell populations could be transformed into each other by ACD (Srinivasan et al., 2016b). Interestingly, compared to the one-way transition from fast-cycling cells to slow-cycling cells in breast cancer, ACD in colorectal cancer establishes a bi-directional transition between the 2 cell populations, thereby sustaining both growth potential and drug resistance of the tumor, enabling rapid response and adaptation of the tumor to a dynamic environment.

Furthermore, tumor cells can generate progenies with survival advantages through ACD, by the selective enrichment of factors that are pro-survival to one of the daughter cells (Lytle et al., 2018). For example, the ATP-binding cassette (ABC) transporters (efflux pumps P glycoprotein 1, also known as ABCB1; and ABC subfamily member 2 (ABCG2)) have the ability to nonspecifically scavenge toxic substances; and cells with high levels of ABC transporters are more resistant to cytotoxic chemotherapy. It was found that in primary cell lines derived from neuroblastoma patients, ACD of ABCG2^hi^ABCA3^hi^ tumor cells generated subpopulations that were ABCG2^hi^ABCA3^hi^ and stem cell-like, as well as subpopulations that were more differentiated and ABCG2^low^ABCA3^low^, suggesting that drug pumps were specifically inherited asymmetrically to a subset of daughter cells to maintain the ABCG2^hi^ABCA3^hi^ cell population that was highly drug-resistant (Hirschmann-Jax et al., 2004). Moreover, a study in neuroblastoma found that CSCs enhanced the therapeutic resistance of daughter cells by asymmetrically co-enriching EGFR and nerve growth factor receptor (p75NTR) in one of the two progeny cells; both receptors in activated state prevent cells from differentiation and enhance the self-renewal capacity of daughter cells (Hitomi et al., 2021).

ACD can also generate and maintain stem cell-like populations with temporary self-renewal capability. Granit et al. discovered a link between asymmetric cell division and the generation of progenitor-like triple negative breast cancer (TNBC) cells. By staining a group of basal-like breast tumors for the basal cell cytokeratin K14, the luminal cytokeratin K18, and the mesenchymal marker vimentin (VIM), Granit found that there were three prominent subpopulations in the tumor samples of invasive TNBC: K14^+^K18^+^, K18^+^ and K18^+^VIM^+^, where the K14^+^K18^+^ subpopulation was luminal progenitor-like and highly tumorigenic. Importantly, they found that progenitor-like K14^+^K18^+^ cells and luminal-like K14^-^K18^+^ were able to convert to each other through undergoing ACD. Adjusting the proportion of progenitor-like K14^+^ cells in TNBC tumors by modulating modes of cell division is an important strategy for promoting drug resistance and progression of TNBC (Granit et al., 2018; Ragoussis, 2018). As more and more evidence implicates the contribution of progenitor cells to the progression of tumor malignancy, it is particularly important to identify progenitor-like cells within tumors and to explore the molecular mechanisms underlying ACD that gives rise to progenitor-like tumor cells.

## Discussion

Since the discovery of ACD and the conserved pathways associated with it, much effort has been devoted to studying how ACD plays a role in various biological processes. In the past 10 years, there has been tremendous progress made in our understanding of the connection between ACD and cancer. The development of high-throughput sequencing, lineage tracing, and other technologies has opened up a new chapter in the study of the intercellular heterogeneity of tumors (Hajirasouliha et al., 2014; Marjanovic et al., 2020); and ACD as an important source of tumor heterogeneity has attracted unprecedented attention. People have begun to explore the biological significance of ACD-related mechanisms in generating heterogeneity. To date, researchers have discovered specific mechanisms of ACD under pathological conditions that are used by tumor cells to generate populations with different properties to enrich the intratumoral heterogeneity (Armah, 2010; Pei and Wechsler-Reya, 2010; Granit et al., 2018; Ragoussis, 2018; Hitomi et al., 2021). These findings may provide new ideas for the search of novel therapeutic targets for the treatment of cancer.

Although we have gained some insights into the relationship between ACD and cancer progression, many questions remain to be answered. First of all, the search for new cell types, cellular components, and molecules that can participate in ACD is still on-going. In recent years, asymmetry at the epigenetic level has attracted much attention, as it may provide new insights into tumor heterogeneity at the epigenetic level (Wooten et al., 2020; Zion et al., 2020; French and Pauklin, 2021; Zion and Chen, 2021). Accumulating evidence indicates that progenitor cells may be the origin of certain types of tumors; however, our knowledge of ACD in these cell types is very limited. As for the regulation of ACD, we lack understanding of the difference in regulatory mechanisms of ACD under physiological and pathological conditions at the molecular level. In particular, regulatory pathways that are specifically activated under pathological conditions deserve to be further explored. In addition, a potential link between tumor metastasis and ACD remains to be elucidated, as a few studies have implicated the association between epithelial-mesenchymal transition (EMT) and ACD. A study in non-small cell lung cancer (NSCLC) cells has shown that signaling activities of the aPKC polarity pathway, a core pathway of ACD, is sufficient to prevent EMT (Gunaratne et al., 2013). In turn, in mouse mammary glands, EMT was found to induce ACD for the enrichment and maintenance of the pool of mammary stem cells (Wu et al., 2019). These findings suggest that ACD and EMT may cooperate to facilitate metastasis of tumor cells.

The heterogeneous population of tumor cells generated by ACD is one of the many factors leading to drug resistance and recurrence of tumors. Gaining more knowledge on how ACD plays a role in cancer development will be of great significance for the understanding of tumor malignancy and the search for potential therapeutic strategies. Inducing ACD of CSCs as a differentiation strategy was initially applied to the treatment of patients with simple acute promyelocytic leukemia (APL) in clinic and achieved some success; and this strategy has been tested for the treatment of solid tumors in recent years (de Thé, 2018). A major advantage of this treatment is its low toxicity compared to traditional chemotherapy and radiotherapy, as it does not kill cells directly. However, the potential risks of these therapeutic strategies are poorly understood. Therefore, thoroughly dissecting the mechanistic details of ACD among different cancer types is essential for further development of therapeutic strategies targeting ACD in cancer.

## References

[B1] Ali S. A. Justilien V. Jamieson L. Murray N. R. Fields A. P. (2016). Protein Kinase Cι Drives a NOTCH3-dependent Stem-like Phenotype in Mutant KRAS Lung Adenocarcinoma. Cancer Cell 29, 367–378. 10.1016/j.ccell.2016.02.012 26977885 PMC4795153

[B2] Armah H. B. (2010). Malignant Astrocytomas Originate from Neural Stem/Progenitor Cells in a Somatic Tumor Suppressor Mouse Model. Yearb. Pathology Laboratory Med. 2010, 171–172. 10.1016/s1077-9108(09)79438-1

[B3] Bai S. Ingram P. Chen Y.-C. Deng N. Pearson A. Niknafs Y. S. (2016). EGFL6 Regulates the Asymmetric Division, Maintenance, and Metastasis of ALDH+ Ovarian Cancer Cells. Cancer Res. 76, 6396–6409. 10.1158/0008-5472.CAN-16-0225 27803106 PMC5120866

[B4] Batlle E. Clevers H. (2017). Cancer Stem Cells Revisited. Nat. Med. 23, 1124–1134. 10.1038/nm.4409 28985214

[B5] Bello B. C. Izergina N. Caussinus E. Reichert H. (2008). Amplification of Neural Stem Cell Proliferation by Intermediate Progenitor Cells in Drosophila Brain Development. Neural Dev. 3, 5. 10.1186/1749-8104-3-5 18284664 PMC2265709

[B6] Betschinger J. Mechtler K. Knoblich J. A. (2006). Asymmetric Segregation of the Tumor Suppressor Brat Regulates Self-Renewal in Drosophila Neural Stem Cells. Cell 124, 1241–1253. 10.1016/j.cell.2006.01.038 16564014

[B7] Bowman S. K. Rolland V. Betschinger J. Kinsey K. A. Emery G. Knoblich J. A. (2008). The Tumor Suppressors Brat and Numb Regulate Transit-Amplifying Neuroblast Lineages in Drosophila. Dev. Cell 14, 535–546. 10.1016/j.devcel.2008.03.004 18342578 PMC2988195

[B8] Bu P. Wang L. Chen K.-Y. Srinivasan T. Murthy P. K. L. Tung K.-L. (2016). A miR-34a-Numb Feedforward Loop Triggered by Inflammation Regulates Asymmetric Stem Cell Division in Intestine and Colon Cancer. Cell Stem Cell 18, 189–202. 10.1016/j.stem.2016.01.006 26849305 PMC4751059

[B9] Cabrera M. C. Hollingsworth R. E. Hurt E. M. (2015). Cancer Stem Cell Plasticity and Tumor Hierarchy. Wjsc 7, 27–36. 10.4252/wjsc.v7.i1.27 25621103 PMC4300934

[B10] Castro-Oropeza R. Melendez-Zajgla J. Maldonado V. Vazquez-Santillan K. (2018). The Emerging Role of lncRNAs in the Regulation of Cancer Stem Cells. Cell Oncol. 41, 585–603. 10.1007/s13402-018-0406-4 PMC12995221 30218296

[B11] Chang W. Wang H. Kim W. Liu Y. Deng H. Liu H. (2020). Hormonal Suppression of Stem Cells Inhibits Symmetric Cell Division and Gastric Tumorigenesis. Cell Stem Cell 26, 739–754. 10.1016/j.stem.2020.01.020 32142681 PMC7214188

[B12] Choi H. Y. Siddique H. R. Zheng M. Kou Y. Yeh D.-W. Machida T. (2020). p53 Destabilizing Protein Skews Asymmetric Division and Enhances NOTCH Activation to Direct Self-Renewal of TICs. Nat. Commun. 11, 3084. 10.1038/s41467-020-16616-8 32555153 PMC7299990

[B13] Cicalese A. Bonizzi G. Pasi C. E. Faretta M. Ronzoni S. Giulini B. (2009). The Tumor Suppressor P53 Regulates Polarity of Self-Renewing Divisions in Mammary Stem Cells. Cell 138, 1083–1095. 10.1016/j.cell.2009.06.048 19766563

[B14] Damodaran A. P. Vaufrey L. Gavard O. Prigent C. (2017). Aurora A Kinase Is a Priority Pharmaceutical Target for the Treatment of Cancers. Trends Pharmacol. Sci. 38, 687–700. 10.1016/j.tips.2017.05.003 28601256

[B15] de Thé H. (2018). Differentiation Therapy Revisited. Nat. Rev. Cancer 18, 117–127. 10.1038/nrc.2017.103 29192213

[B16] Dey-Guha I. Wolfer A. Yeh A. C. G. Albeck J. Darp R. Leon E. (2011). Asymmetric Cancer Cell Division Regulated by AKT. Proc. Natl. Acad. Sci. U.S.A. 108, 12845–12850. 10.1073/pnas.1109632108 21757645 PMC3150943

[B17] Donnenberg V. S. Donnenberg A. D. (2005). Multiple Drug Resistance in Cancer Revisited: the Cancer Stem Cell Hypothesis. J. Clin. Pharmacol. 45, 872–877. 10.1177/0091270005276905 16027397

[B18] French R. Pauklin S. (2021). Epigenetic Regulation of Cancer Stem Cell Formation and Maintenance. Int. J. Cancer 148, 2884–2897. 10.1002/ijc.33398 33197277 PMC8246550

[B19] Gerdes M. J. Sood A. Sevinsky C. Pris A. D. Zavodszky M. I. Ginty F. (2014). Emerging Understanding of Multiscale Tumor Heterogeneity. Front. Oncol. 4, 366. 10.3389/fonc.2014.00366 25566504 PMC4270176

[B20] Gómez-López S. Lerner R. G. Petritsch C. (2014). Asymmetric Cell Division of Stem and Progenitor Cells during Homeostasis and Cancer. Cell. Mol. Life Sci. 71, 575–597. 10.1007/s00018-013-1386-1 23771628 PMC3901929

[B21] Granit R. Z. Masury H. Condiotti R. Fixler Y. Gabai Y. Glikman T. (2018). Regulation of Cellular Heterogeneity and Rates of Symmetric and Asymmetric Divisions in Triple-Negative Breast Cancer. Cell Rep. 24, 3237–3250. 10.1016/j.celrep.2018.08.053 30232005

[B22] Gunaratne A. Thai B. L. Di Guglielmo G. M. (2013). Atypical Protein Kinase C Phosphorylates Par6 and Facilitates Transforming Growth Factor β-Induced Epithelial-To-Mesenchymal Transition. Mol. Cell Biol. 33, 874–886. 10.1128/MCB.00837-12 23249950 PMC3623079

[B23] Hajirasouliha I. Mahmoody A. Raphael B. J. (2014). A Combinatorial Approach for Analyzing Intra-tumor Heterogeneity from High-Throughput Sequencing Data. Bioinformatics 30, i78–i86. 10.1093/bioinformatics/btu284 24932008 PMC4058927

[B24] Hirschmann-Jax C. Foster A. E. Wulf G. G. Nuchtern J. G. Jax T. W. Gobel U. (2004). A Distinct "side Population" of Cells with High Drug Efflux Capacity in Human Tumor Cells. Proc. Natl. Acad. Sci. U.S.A. 101, 14228–14233. 10.1073/pnas.0400067101 15381773 PMC521140

[B25] Hitomi M. Chumakova A. P. Silver D. J. Knudsen A. M. Pontius W. D. Murphy S. (2021). Asymmetric Cell Division Promotes Therapeutic Resistance in Glioblastoma Stem Cells. JCI Insight 6, e130510. 10.1172/jci.insight.130510 PMC7934841 33351787

[B26] Iden S. van Riel W. E. Schäfer R. Song J.-Y. Hirose T. Ohno S. (2012). Tumor Type-dependent Function of the Par3 Polarity Protein in Skin Tumorigenesis. Cancer Cell 22, 389–403. 10.1016/j.ccr.2012.08.004 22975380

[B27] Ito T. Kwon H. Y. Zimdahl B. Congdon K. L. Blum J. Lento W. E. (2010). Regulation of Myeloid Leukaemia by the Cell-Fate Determinant Musashi. Nature 466, 765–768. 10.1038/nature09171 20639863 PMC2918284

[B28] Izumi Y. Ohta N. Hisata K. Raabe T. Matsuzaki F. (2006). Drosophila Pins-Binding Protein Mud Regulates Spindle-Polarity Coupling and Centrosome Organization. Nat. Cell Biol. 8, 586–593. 10.1038/ncb1409 16648846

[B29] Kahney E. W. Ranjan R. Gleason R. J. Chen X. (2017). Symmetry from Asymmetry or Asymmetry from Symmetry? Cold Spring Harb. Symp. Quant. Biol. 82, 305–318. 10.1101/sqb.2017.82.034272 29348326 PMC6245645

[B30] Kasper S. (2008). Exploring the Origins of the Normal Prostate and Prostate Cancer Stem Cell. Stem Cell Rev. 4, 193–201. 10.1007/s12015-008-9033-1 18563640 PMC11075662

[B31] Keysar S. B. Le P. N. Miller B. Jackson B. C. Eagles J. R. Nieto C. (2017). Regulation of Head and Neck Squamous Cancer Stem Cells by PI3K and SOX2. JNCI J. Natl. Cancer Inst. 109, djw189. 10.1093/jnci/djw189 PMC5025278 27634934

[B32] Kim H. Ronai Z. e. A. (2018). Rewired Notch/p53 by Numb'ing Mdm2. J. Cell Biol. 217, 445–446. 10.1083/jcb.201712007 29339436 PMC5800819

[B33] Klezovitch O. Fernandez T. E. Tapscott S. J. Vasioukhin V. (2004). Loss of Cell Polarity Causes Severe Brain Dysplasia in Lgl1 Knockout Mice. Genes Dev. 18, 559–571. 10.1101/gad.1178004 15037549 PMC374237

[B34] Knoblich J. A. (2010). Asymmetric Cell Division: Recent Developments and Their Implications for Tumour Biology. Nat. Rev. Mol. Cell Biol. 11, 849–860. 10.1038/nrm3010 21102610 PMC3941022

[B35] Lee C.-Y. Robinson K. J. Doe C. Q. (2006). Lgl, Pins and aPKC Regulate Neuroblast Self-Renewal versus Differentiation. Nature 439, 594–598. 10.1038/nature04299 16357871

[B36] Lee C.-Y. Wilkinson B. D. Siegrist S. E. Wharton R. P. Doe C. Q. (2006). Brat Is a Miranda Cargo Protein that Promotes Neuronal Differentiation and Inhibits Neuroblast Self-Renewal. Dev. Cell 10, 441–449. 10.1016/j.devcel.2006.01.017 16549393

[B37] Lee G. R Hall R. 3rd Ahmed A. U. (2016). Cancer Stem Cells: Cellular Plasticity, Niche, and its Clinical Relevance. J. Stem Cell Res. Ther. 06, 363. 10.4172/2157-7633.1000363 PMC5123595 27891292

[B38] Liu Y. Siles L. Lu X. Dean K. C. Cuatrecasas M. Postigo A. (2018). Mitotic Polarization of Transcription Factors during Asymmetric Division Establishes Fate of Forming Cancer Cells. Nat. Commun. 9, 2424. 10.1038/s41467-018-04663-1 29930325 PMC6013470

[B39] Lytle N. K. Barber A. G. Reya T. (2018). Stem Cell Fate in Cancer Growth, Progression and Therapy Resistance. Nat. Rev. Cancer 18, 669–680. 10.1038/s41568-018-0056-x 30228301 PMC8388042

[B40] Majumdar S. Liu S. T. Liu S.-T. (2020). Cell Division Symmetry Control and Cancer Stem Cells. AIMS Mol. Sci. 7, 82–101. 10.3934/molsci.2020006 32953979 PMC7500705

[B41] Marjanovic N. D. Hofree M. Chan J. E. Canner D. Wu K. Trakala M. (2020). Emergence of a High-Plasticity Cell State during Lung Cancer Evolution. Cancer Cell 38, 229–246 e13. 10.1016/j.ccell.2020.06.012 32707077 PMC7745838

[B42] Mccaffrey L. M. Macara I. G. (2009). The Par3/aPKC Interaction Is Essential for End Bud Remodeling and Progenitor Differentiation during Mammary Gland Morphogenesis. Genes Dev. 23, 1450–1460. 10.1101/gad.1795909 19528321 PMC2701573

[B43] Mccaffrey L. M. Montalbano J. Mihai C. Macara I. G. (2012). Loss of the Par3 Polarity Protein Promotes Breast Tumorigenesis and Metastasis. Cancer Cell 22, 601–614. 10.1016/j.ccr.2012.10.003 23153534 PMC3500525

[B44] Mcgill M. A. Mcglade C. J. (2003). Mammalian Numb Proteins Promote Notch1 Receptor Ubiquitination and Degradation of the Notch1 Intracellular Domain. J. Biol. Chem. 278, 23196–23203. 10.1074/jbc.M302827200 12682059

[B45] Mizukawa B. O’Brien E. Moreira D. C. Wunderlich M. Hochstetler C. L. Duan X. (2017). The Cell Polarity Determinant CDC42 Controls Division Symmetry to Block Leukemia Cell Differentiation. Blood 130, 1336–1346. 10.1182/blood-2016-12-758458 28778865 PMC5600140

[B46] Morrison S. J. Kimble J. (2006). Asymmetric and Symmetric Stem-Cell Divisions in Development and Cancer. Nature 441, 1068–1074. 10.1038/nature04956 16810241

[B47] Neumüller R. A. Knoblich J. A. (2009). Dividing Cellular Asymmetry: Asymmetric Cell Division and its Implications for Stem Cells and Cancer. Genes Dev. 23, 2675–2699. 10.1101/gad.1850809 19952104 PMC2788323

[B48] Pajuelo-Lozano N. Alcalá S. Sainz B. Jr. Perona R. Sanchez-Perez I. (2020). Targeting MAD2 Modulates Stemness and Tumorigenesis in Human Gastric Cancer Cell Lines. Theranostics 10, 9601–9618. 10.7150/thno.49270 32863948 PMC7449921

[B49] Pece S. Serresi M. Santolini E. Capra M. Hulleman E. Galimberti V. (2004). Loss of Negative Regulation by Numb over Notch Is Relevant to Human Breast Carcinogenesis. J. Cell Biol. 167, 215–221. 10.1083/jcb.200406140 15492044 PMC2172557

[B50] Pece S. Tosoni D. Confalonieri S. Mazzarol G. Vecchi M. Ronzoni S. (2010). Biological and Molecular Heterogeneity of Breast Cancers Correlates with Their Cancer Stem Cell Content. Cell 140, 62–73. 10.1016/j.cell.2009.12.007 20074520

[B51] Pei Y. Wechsler-Reya R. J. (2010). A Malignant Oligarchy: Progenitors Govern the Behavior of Oligodendrogliomas. Cancer Cell 18, 546–547. 10.1016/j.ccr.2010.11.031 21156279 PMC3012268

[B52] Ragoussis J. (2018). Regulators of Asymmetric Cell Division in Breast Cancer. Trends Cancer 4, 798–801. 10.1016/j.trecan.2018.10.009 30470301

[B53] Rossi A. M. Desplan C. (2017). Asymmetric Notch Amplification to Secure Stem Cell Identity. Dev. Cell 40, 513–514. 10.1016/j.devcel.2017.03.010 28350981 PMC5490801

[B54] Santoro A. Vlachou T. Carminati M. Pelicci P. G. Mapelli M. (2016). Molecular Mechanisms of Asymmetric Divisions in Mammary Stem Cells. EMBO Rep. 17, 1700–1720. 10.15252/embr.201643021 27872203 PMC5283594

[B55] Sell S. (2007). Cancer and Stem Cell Signaling: a Guide to Preventive and Therapeutic Strategies for Cancer Stem Cells. Stem Cell Rev. 3, 1–6. 10.1007/s12015-007-0015-5 17873376

[B56] Sell S. (2010). On the Stem Cell Origin of Cancer. Am. J. Pathology 176, 2584–2594. 10.2353/ajpath.2010.091064 PMC2877820 20431026

[B57] Singh A. K. Arya R. K. Maheshwari S. Singh A. Meena S. Pandey P. (2015). Tumor Heterogeneity and Cancer Stem Cell Paradigm: Updates in Concept, Controversies and Clinical Relevance. Int. J. Cancer 136, 1991–2000. 10.1002/ijc.28804 24615680

[B58] Singh A. Settleman J. (2010). EMT, Cancer Stem Cells and Drug Resistance: an Emerging axis of Evil in the War on Cancer. Oncogene 29, 4741–4751. 10.1038/onc.2010.215 20531305 PMC3176718

[B59] Srinivasan T. Than E. B. Bu P. Tung K.-L. Chen K.-Y. Augenlicht L. (2016). Notch Signalling Regulates Asymmetric Division and Inter-conversion between Lgr5 and Bmi1 Expressing Intestinal Stem Cells. Sci. Rep. 6, 26069. 10.1038/srep26069 27181744 PMC4867651

[B60] Srinivasan T. Walters J. Bu P. Than E. B. Tung K.-L. Chen K.-Y. (2016). NOTCH Signaling Regulates Asymmetric Cell Fate of Fast- and Slow-Cycling Colon Cancer-Initiating Cells. Cancer Res. 76, 3411–3421. 10.1158/0008-5472.CAN-15-3198 27197180 PMC4891252

[B61] Stypulkowski E. Asangani I. A. Witze E. S. (2018). The Depalmitoylase APT1 Directs the Asymmetric Partitioning of Notch and Wnt Signaling during Cell Division. Sci. Signal. 11, eaam8705. 10.1126/scisignal.aam8705 29295957 PMC5914505

[B62] Sugioka K. Fielmich L.-E. Mizumoto K. Bowerman B. Van Den Heuvel S. Kimura A. (2018). Tumor Suppressor APC Is an Attenuator of Spindle-Pulling Forces during *C. elegans* Asymmetric Cell Division. Proc. Natl. Acad. Sci. U.S.A. 115, E954–E963. 10.1073/pnas.1712052115 29348204 PMC5798331

[B63] Tosoni D. Zecchini S. Coazzoli M. Colaluca I. Mazzarol G. Rubio A. (2015). The Numb/p53 Circuitry Couples Replicative Self-Renewal and Tumor Suppression in Mammary Epithelial Cells. J. Cell Biol. 211, 845–862. 10.1083/jcb.201505037 26598619 PMC4657167

[B64] Varga J. Greten F. R. (2017). Cell Plasticity in Epithelial Homeostasis and Tumorigenesis. Nat. Cell Biol. 19, 1133–1141. 10.1038/ncb3611 28945230

[B65] Viale A. Pettazzoni P. Lyssiotis C. A. Ying H. Sánchez N. Marchesini M. (2014). Oncogene Ablation-Resistant Pancreatic Cancer Cells Depend on Mitochondrial Function. Nature 514, 628–632. 10.1038/nature13611 25119024 PMC4376130

[B66] Wirtz-Peitz F. Nishimura T. Knoblich J. A. (2008). Linking Cell Cycle to Asymmetric Division: Aurora-A Phosphorylates the Par Complex to Regulate Numb Localization. Cell 135, 161–173. 10.1016/j.cell.2008.07.049 18854163 PMC2989779

[B67] Wooten M. Ranjan R. Chen X. (2020). Asymmetric Histone Inheritance in Asymmetrically Dividing Stem Cells. Trends Genet. 36, 30–43. 10.1016/j.tig.2019.10.004 31753528 PMC6925335

[B68] Wu M.-J. Chen Y.-S. Kim M. R. Chang C.-C. Gampala S. Zhang Y. (2019). Epithelial-Mesenchymal Transition Directs Stem Cell Polarity via Regulation of Mitofusin. Cell Metab. 29, 993–1002 e6. 10.1016/j.cmet.2018.11.004 30527740

[B69] Zimdahl B. Ito T. Blevins A. Bajaj J. Konuma T. Weeks J. (2014). Lis1 Regulates Asymmetric Division in Hematopoietic Stem Cells and in Leukemia. Nat. Genet. 46, 245–252. 10.1038/ng.2889 24487275 PMC4267534

[B70] Zion E. Chen X. (2021). Breaking Symmetry: The Asymmetries in Epigenetic Inheritance. Biochem. (Lond) 43, 14–19. 10.1042/bio_2020_110 34354328 PMC8330550

[B71] Zion E. H. Chandrasekhara C. Chen X. (2020). Asymmetric Inheritance of Epigenetic States in Asymmetrically Dividing Stem Cells. Curr. Opin. Cell Biol. 67, 27–36. 10.1016/j.ceb.2020.08.003 32871437 PMC7736099

